# The role and regulation of Rab40b–Tks5 complex during invadopodia formation and cancer cell invasion

**DOI:** 10.1242/jcs.193904

**Published:** 2016-12-01

**Authors:** Abitha Jacob, Erik Linklater, Brian A. Bayless, Traci Lyons, Rytis Prekeris

**Affiliations:** 1 Department of Cell and Developmental Biology, School of Medicine, Anschutz Medical Campus, University of Colorado Denver, Aurora, CO 80045, USA; 2 Department of Medicine/Division of Medical Oncology, School of Medicine, Anschutz Medical Campus, University of Colorado Denver, Aurora, CO 80045, USA

**Keywords:** MMP2, MMP9, Rab40b, Tks5, Invadopodia, Metastasis

## Abstract

Invadopodia formation and extracellular matrix degradation are key events during cancer cell invasion, yet little is known about mechanisms mediating these processes. Here, we report that Rab40b plays a key role in mediating invadopodia function during breast cancer cell invasion. We also identify Tks5 (also known as SH3PXD2A), a known Src kinase substrate, as a new Rab40b effector protein and show that Tks5 functions as a tether that mediates Rab40b-dependent targeting of transport vesicles containing MMP2 and MMP9 to the extending invadopodia. Importantly, we also demonstrate that Rab40b and Tks5 levels are regulated by known tumor suppressor microRNA miR-204. This is the first study that identifies a new Rab40b–Tks5- and miR-204-dependent invadopodia transport pathway that regulates MMP2 and MMP9 secretion, and extracellular matrix remodeling during cancer progression.

## Abstract (precis)

**Highlighted Article:** Rab40b plays a key role in mediating invadopodia function during breast cancer cell invasion by binding to Tks5 and functioning as a tether mediating MMP2 and MMP9 targeting to the extending invadopodia.

## INTRODUCTION

The first event that occurs during epithelial cancer metastasis is the breach of the basement membrane, which leads to cell invasion. The basement membrane is a highly specialized extracellular matrix (ECM) that provides structural support to tissues by surrounding all epithelium and endothelium (LeBleu et al., 2007). Breaching the basement membrane is facilitated by actin-rich cellular protrusions known as invadopodia. These invasive matrix-degrading structures were originally identified in cells transformed with activated Src (David-Pfeuty and Singer, 1980; Tarone et al., 1985) and are known to play an important role during cancer cell invasion.

The matrix degradation activity of invadopodia is attributed to the targeted secretion of matrix-degrading enzymes such as matrix metalloproteinases (MMPs). MMPs are known for their ability to degrade several components of the ECM and are important for normal processes such as tissue remodeling and wound healing. Aberrant expression and secretion of MMPs has been correlated with promotion of metastasis. In particular, MMP2, MMP9 and MMP14 have all been shown to promote cancer progression due to their ability to degrade basement membrane components. Importantly, MMP2, MMP9 and MMP14 are enriched at the invadopodia and are required for cancer metastasis (Ala-aho and Kahari, 2005; Bjorklund and Koivunen, 2005; Hofmann et al., 2005; Kerkela and Saarialho-Kere, 2003; Lochter et al., 1998; Mook et al., 2004; Wagenaar-Miller et al., 2004). Owing to the importance of MMPs in cancer progression, much work has been focused on identifying the mechanisms governing targeted MMP secretion. Thus far, it has been shown that accumulation of MMP14 at the invadopodia is regulated by endocytic uptake and exocytosis pathways. Additionally, it has been shown that MMP14 intracellular traffic is mediated by several endocytic transport proteins such as Rab8, the exocyst complex, VAMP7 and Tks4 (also known as SH3PXD2B) (reviewed in Jacob and Prekeris, 2015; Castro-Castro et al., 2016).

In contrast to MMP14, the factors governing MMP2 and MMP9 targeting to the invadopodia remain largely unknown. It has been shown that MMP2 and MMP9 are not transported to the invadopodia by endosomes, but instead are targeted directly from the Golgi (Jacob et al., 2013) through microtubule motors like kinesin and by actin regulators like cortactin, thus demonstrating that MMP14, and MMP2 and MMP9 are targeted to invadopodia through two distinct membrane transport pathways.

Our recent work identified Rab40b GTPase as a protein required for MMP2 and MMP9 secretion from the invadopodia in breast cancer cells (Jacob et al., 2013). We also demonstrated that Rab40b is required for cancer cell invasion *in vitro* (Jacob et al., 2013). However, how Rab40b regulates targeted MMP2 and MMP9 secretion and localized ECM remodeling remains to be understood, and the machinery that regulates levels of Rab40b in cancer cells is also unknown. Although we have shown that Rab40b is required for MMP2 and MMP9 secretion *in vitro*, it remains unclear whether Rab40b mediates MMP2 and MMP9 secretion during breast cancer cell invasion and metastasis *in vivo.* These questions are the focus of this study. Here, we show that Rab40b is required for breast tumor growth and metastasis *in vivo* and that Rab40b levels are increased in metastatic breast cancers. Given that all Rab GTPases function by binding to various regulatory factors, we also screened for Rab40b-binding proteins and identified tyrosine kinase substrate 5 (Tks5, also known as SH3PXD2A) as a Rab40b-binding partner. Importantly, Tks5 is a large scaffolding protein that is phosphorylated by Src kinase and is required for the formation and maturation of invadopodia (Courtneidge et al., 2005; Sharma et al., 2013). Here, we characterize biochemical and structural properties of Rab40b and Tks5 binding and show that Tks5 functions as a tether mediating the targeting of transport vesicles containing MMP2, MMP9 and Tks5 to the extending invadopodia.

Given that Rab40b and Tks5 are upregulated in metastatic breast cancer cells, we also investigated the regulation of Rab40b expression. We demonstrate that miR-204, a known tumor suppressor microRNA, regulates the expression of both Rab40b and Tks5. Although miR-204 has been previously shown to suppress cancer metastasis, the mechanism and the downstream targets that mediate the anti-invasive miR-204 effects remained unclear. Here, we propose that miR-204 functions as a tumor suppressor by downregulating Rab40b and Tks5 levels, thus directly inhibiting invadopodia extension and localized ECM remodeling. Taken together, this study describes and characterizes a new Rab40b–Tks5-dependent transport pathway that mediates invadopodia extension and function during breast cancer metastasis. Additionally, we show that miR-204 acts as a tumor suppressor by regulating Rab40b and Tks5 expression and consequently inhibiting MMP2 and MMP9 targeting, which leads to a decrease in invadopodia-associated ECM degradation.

## RESULTS

### Rab40b is required for breast cancer cell invasion and invadopodia extension

**Fig. 1.**
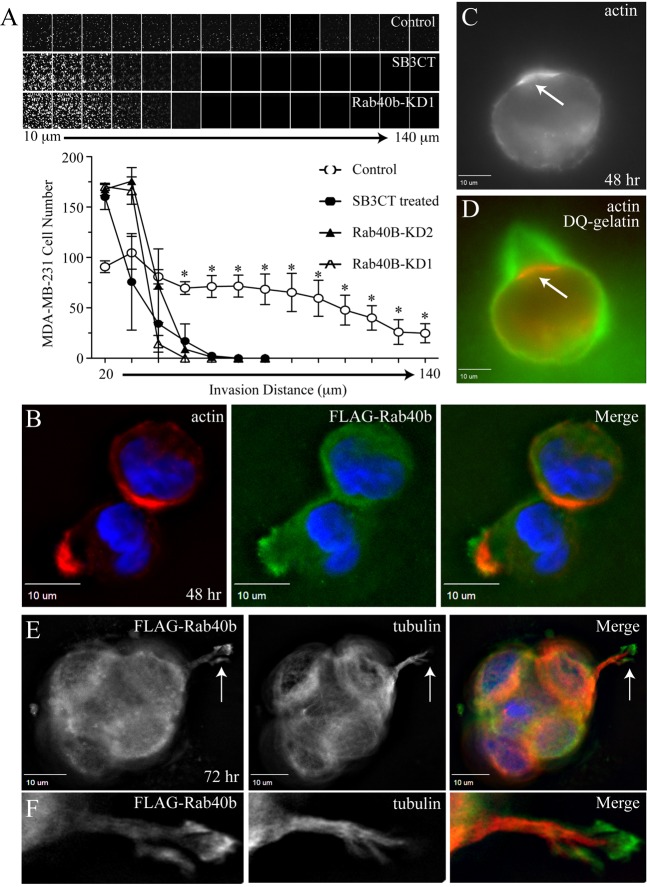
**Rab40b localizes to the invadopodia and regulates cancer cell invasion.** (A) Control MDA-MB-231 cells or MDA-MB-231 cells stably expressing Rab40b shRNAs (KD1 or KD2), were plated on a transwell filter containing a gelatin plug and allowed to invade towards a growth-factor-rich gradient for 5 days. As positive control, one set of wild-type MDA-MB-231 cells were also treated with SB3CT (an MMP2 and MMP9 inhibitor). The cells were stained with Calcein and imaged at 10-µm steps to measure distance of invasion. Data shown underneath are the mean±s.d. of three independent experiments. For every data point, cells in 15 randomly chosen fields were counted. *P<0.005, control data points are significantly different from cells treated with SB3CT or expressing Rab40b shRNA (two-tailed Student's *t*-test). (B,E,F) MDA-MB-231 cells stably expressing FLAG–Rab40b were mixed with Matrigel and collagen I, embedded on a 3D chamber slide and incubated for 48 h (B) and 72 h (E,F). The spheroids were stained with anti-tubulin and anti-FLAG antibodies. (C,D) MDA-MB-231 cells were embedded in a Matrigel, collagen I and DQ-gelatin mixture and incubated for 48 h. The arrows indicate forming invadopodia.

Recently, we identified Rab40b as a GTPase that is required for MMP2 and MMP9 secretion and invadopodia-associated ECM degradation in MDA-MB-231 cells cultured on two-dimensional (2D) surfaces (Jacob et al., 2013). However, it is becoming widely accepted that 2D invadopodia formation assays might not always allow the testing of all the *in vivo* aspects of cell invasion machinery. Thus, to further define the role of Rab40b in mediating cancer cell invasion through the ECM, we used three-dimensional (3D) invasion assays, which more closely simulate the *in vivo* environment (Caswell et al., 2007; von Thun et al., 2012). Such 3D invasion assays provide more information as they allow the measurement of the dynamics and invasive capacities of individual cells. To analyze the function of Rab40b in mediating MMP2 and MMP9 secretion in 3D invasion assays, we replaced Matrigel with 2.5% cross-linked gelatin supplemented with 10 µg/ml fibronectin. We chose to use gelatin because it is a known MMP2 and MMP9 substrate. Furthermore cross-linked gelatin creates a stiffer 3D matrix as compared to Matrigel (Artym et al., 2015; Van Goethem et al., 2010). Higher ECM stiffness has been shown to induce invadopodia formation and also correlate with poor breast cancer prognosis (Chaudhuri et al., 2014). To test whether Rab40b knockdown affects cell invasion through stiff ECM, we generated MDA-MB-231 cell lines stably expressing either non-targeting short hairpin RNA (shRNA) (control) or two different Rab40b shRNAs, named KD1 (80% knockdown) and KD2 (50% knockdown) (for quantification see Fig. S1A) and found that depletion of Rab40b decreased MDA-MB-231 cell invasion (Fig. 1A). Importantly, treatment of MDA-MB-231 cells with SB3CT, a known specific MMP2 and MMP9 inhibitor, caused a comparable decrease in invasion (Fig. 1A).

**Fig. 2.**
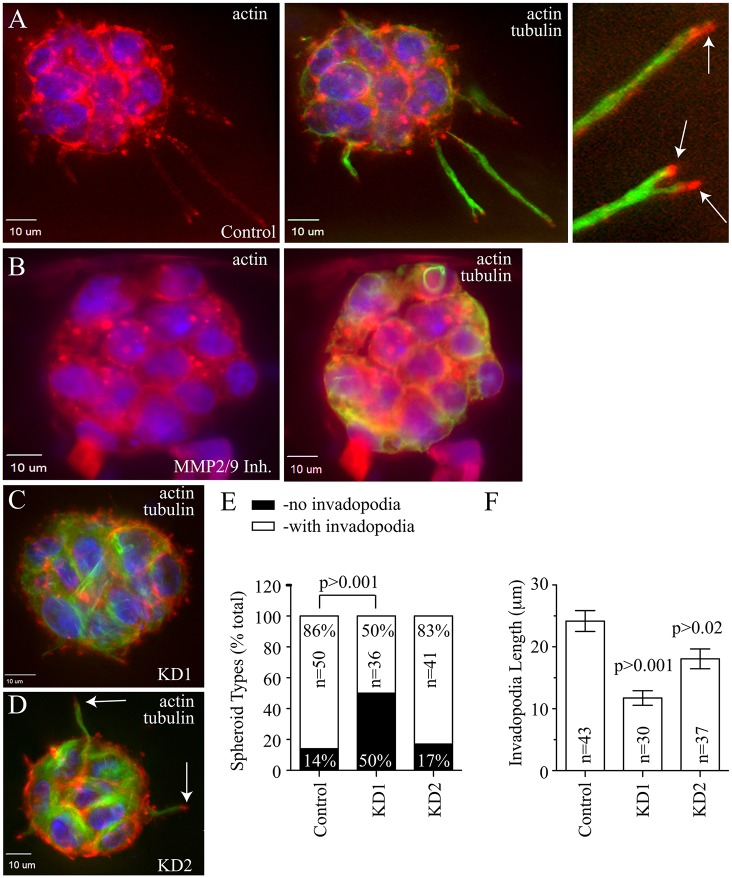
**Rab40b is required for invadopodia extension and maturation.** (A–D) MDA-MB-231 cells, wild-type (A,B) or stably expressing Rab40b shRNA (KD1 or KD2; C,D) were embedded in Matrigel+collagen I mixture and grown for 4 days. The spheroids were then fixed and stained with anti-tubulin (green) and phalloidin (red) antibodies to mark mature invadopodia. In B, wild-type spheroids were treated with 50 µM of MMP2 and MMP9 inhibitor (Inh.) SB3CT for the last 48 h of 4 day growth. The arrows in A and D indicate invadopodia. (E,F) Invadopodia length (F) and number of spheroids with invadopdodia (E) were analyzed for each set of cells. Data shown are the mean±s.d. from three independent experiments. *n* is the total number of spheroids analyzed. *P*-values given are compared to control and calculated with a two-tailed Student's *t*-test.

We have previously shown that Rab40b is required for invadopodia-dependent ECM degradation in 2D *in situ* zymography assays while having no effect on invadopodia formation (Jacob et al., 2013). In 2D assays, cells form invadopodia that lack the physical space to develop into fully mature invasive structures due to the thin layer of matrix. To determine whether Rab40b has a role in invadopodia formation and maturation, we embedded MDA-MB-231 cells expressing FLAG–Rab40b (MDA-MB-231-FLAG-Rab40b) in Matrigel supplemented with 1 mg/ml collagen I. Similar to gelatin matrices, Matrigel and collagen form a stiff matrix that leads to the formation of MDA-MB-231 spheroids with well-defined invadopodia-like structures. By 48 h post embedding, many cells had already formed well-defined and actin-rich invadopodia precursors (Fig. 1B). Even at these early stages of invadopodia formation, some Rab40b accumulation could be observed at the tips of these actin-rich structures (Fig. 1B). These invadopodia precursors also showed ECM degradation activity as detected by DQ-gelatin around the actin-enriched bud (Fig. 1C,D). At 4 days post-embedding, the invadopodia precursors extended into long mature invadopodia as marked by the presence of microtubules (Figs 1E and 2A), a known marker for mature invadopodia (Schoumacher et al., 2010). The tips of these invadopodia-like structures contained actin (Fig. 2A) and were also highly enriched in FLAG–Rab40b (Fig. 1F). The formation of these invadopodia was completely blocked if spheroids were treated with the MMP2 and MMP9 inhibitor SB3CT, demonstrating that formation and extension of invadopodia in 3D spheroid culture is dependent on MMP2 and MMP9 secretion (Fig. 2B).

To further test whether Rab40b is required for invadopodia maturation, we next analyzed 4-day spheroids of wild-type MDA-MB-231 and cells stably expressing Rab40b shRNAs. As shown in Fig. 2C–F, Rab40b knockdown resulted in a decreased length of mature invadopodia. Interestingly, whereas spheroids from both Rab40b-knockdown cell lines had shorter invadopodia-like structures (Fig. 2F), the Rab40b KD1 line had fewer spheroids with invadopodia (Fig. 2E). This difference in phenotype is likely due to the fact that the KD1 cell line has better Rab40b knock-down as compared to KD2 (80% and 50% depletion, respectively; Fig. S1A). After incubating MDA-MB-231 spheroids for 7 days, many of the invadopodia developed into invasive chains containing multiple cells migrating out of the primary spheroid (Fig. S1B). Consistent with the proposed role of Rab40b in regulating cancer metastasis, Rab40b depletion decreased the number of these invasive strands (Fig. S1C). Taken together, the data suggest that Rab40b is primarily required for invadopodia extension and ECM degradation, although we cannot fully rule out that Rab40b might also have a role in the formation or stability of early invadopodia precursors.

### Rab40b is required for primary tumor growth and metastasis *in vivo*

**Fig. 3.**
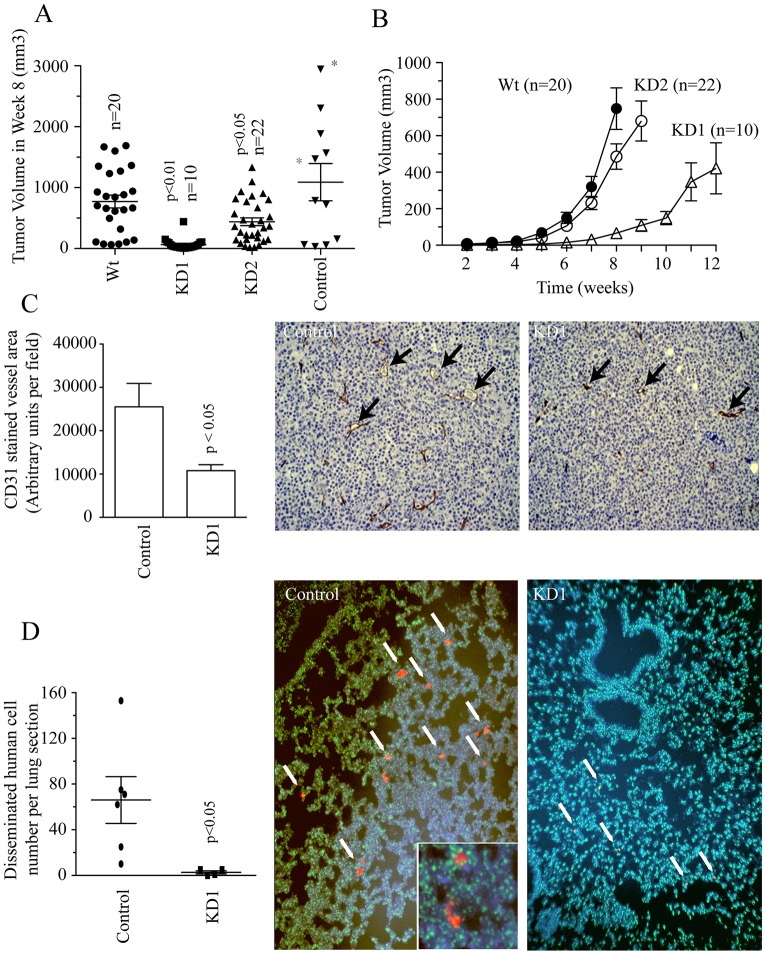
**Rab40b knockdown affects vasculogenesis, primary tumor growth and metastasis *in vivo*.** (A) Wild-type MDA-MB-231 (Wt), shRNA control (control) and Rab40b knockdown (KD1 or KD2) cells were injected in the mammary fat pad of hairless SCID mice. Mice were killed and tumor volumes were measured 8 weeks after injection. 10–25 mice per cell line type were analyzed. Asterisks (*) mark the mice that had to be killed at week 7 due to tumor sizes exceeding guidelines and ulceration. Data shown are the mean±s.d. *n* is the total number of tumors measured. (B) Wild-type (Wt) and Rab40b KD (KD1 or KD2) cells were implanted in the mammary fat pads of hairless SCID mice. 10–25 mice per cell line type were analyzed. Tumor volumes were measured every week and allowed to grow to a final tumor burden of 2 cm^3^. Data shown are mean±s.d. *n* is the total number of tumors analyzed. (C) Tumors isolated 8 weeks after injection from control (wild type) and Rab40b KD1 mice were analyzed through immunohistochemistry. Vessel area was examined by staining tumor samples with anti-CD31 antibodies. 15 random chosen fields in three separate sections were analyzed for each tumor. Data shown are the mean±s.d. derived from three different control and three different KD1 tumors. Arrows in images point to individual CD31-positive vessels. Note the difference in size between vessels in control and KD1 primary tumors. (D) Lungs isolated 8 weeks after injection from control and KD1 mice were analyzed by a dual-color FISH assay. Three lungs were analyzed per each tumor group. 15 randomly chosen fields from five separate sections from every lung were analyzed. The scatterplot shows the average number of metastases in each section per lung, and the mean±s.d. is indicated. Metastatic lesions consisting of human cells are indicated as red clumps and marked with white arrows in a sea of green mouse cells and blue DAPI stain. Each image is representative of the entire tumor group. *P*-values given are compared to control and calculated with a two-tailed Student's *t*-test.

Our previous and current *in vitro* experiments show that Rab40b regulates MMP2 and MMP9 targeting to the invadopodia. In order to determine whether Rab40b has any effect on tumor growth and metastasis *in vivo*, we used SCID mice to perform mammary fat pad injections with either control, KD1 or KD2 MDA-MB-231 cell lines. Surprisingly, at 8 weeks after tumor cell injection, we observed a significant difference in tumor sizes between shRNA control and knockdown mice (Fig. 3A). Furthermore, when tumors were allowed to reach a total tumor burden of 2 cm^3^ in a size-matched study, primary tumor volume was significantly lower in KD1- or KD2-injected mice (Fig. 3B; Fig. S1D), suggesting that Rab40b plays a role in regulating primary tumor growth.

We next sought to explain the decrease in tumor size in Rab40b KD tumors *in vivo*. Given that Rab40b knockdown does not appear to directly affect cell proliferation *in vitro* (Fig. S1E), we tested whether proliferation and/or apoptosis were altered within the primary tumor. To that end, we analyzed primary tumors harvested from control and Rab40b KD mice 8 weeks after injection. Rab40b knockdown did not cause changes in proliferation or apoptosis *in vivo* (Fig. S2A–C). Given that vasculogenesis is vital for tumor growth and progression (Weis and Cheresh, 2011) and MMP2 and MMP9 have been implicated in angiogenesis (Schnaper et al., 1993; Seftor et al., 2001; Itoh et al., 1998; Vu et al., 1998), we next tested whether Rab40b knockdown is required for primary tumor vascularization. As shown in Fig. 3C, Rab40b KD tumors had significantly smaller blood vessels compared to the control. Although there were no significant differences in apoptosis at 8 weeks after injection, we analyzed whether the decrease in vessel density in early tumorigenesis might cause apoptosis during late tumorigenesis, when tumors are allowed to grow to a final total volume of 2 cm^3^. Consistent with the involvement of Rab40b in mediating transport of MMP2 and MMP9, which have been implicated in vasculogenesis, there was a significant increase in apoptosis in large KD tumors as compared to control (Fig. S2D,E), likely caused by the lack of tumor vascularization in early tumorigenesis, which could lead to hypoxia and eventually apoptosis. Taken together, our results suggest that Rab40b might regulate vasculogenesis, thus affecting primary tumor growth.

Given that our initial studies suggested that Rab40b is required for MDA-MB-231 cell invasion *in vitro*, we hypothesized that Rab40b knockdown should also affect breast tumor metastasis *in vivo*. The examination of lungs harvested from control mice 8 weeks after injection, revealed small clumps of disseminated human cells dispersed throughout the lungs (Fig. 3D; human cells in red and marked by arrows). Occasionally, we also observed large clumps of human cells in lungs of control mice (Fig. S1F,G). In contrast, no large clumps were observed at 8 weeks in the lungs of mice injected with Rab40b-KD1 cells. Furthermore, the number of lung micro-metastases was significantly lower in mice injected with Rab40b-KD1 cells (Fig. 3D). Thus, our *in vivo* studies demonstrate that Rab40b promotes tumor metastasis by regulating primary tumor growth as well as invasion of breast cancer cells.

### Tks5 is a Rab40b-binding protein that regulates invadopodia function

Identification of Rab40b effector proteins is a key step to understanding the molecular machinery governing Rab40b function. To identify such effector proteins, we incubated MDA-MB-231 cell lysates with either GST or GST–Rab40b-coated beads. Proteins bound to beads were then analyzed by mass spectrometry. Only proteins that were identified in GST–Rab40b eluates and not present in GST-only eluates were analyzed further. Additionally, we classified all RNA-, DNA- or mitochondria-associated proteins as contaminants. All the remaining proteins were classified as putative Rab40b-interacting proteins and are listed in Fig. S3A. Interestingly, Tks5 was identified as a putative Rab40b-binding protein (Fig. S3A). Tks5 is a known invadopodia protein that has been reported to regulate invadopodia formation and maturation (Courtneidge et al., 2005; Sharma et al., 2013). Furthermore, Tks5 was shown to be required for ECM degradation *in vitro* (Stylli et al., 2009) as well as tumor growth and metastasis *in vivo* (Blouw et al., 2015; Blouw et al., 2008).

**Fig. 4.**
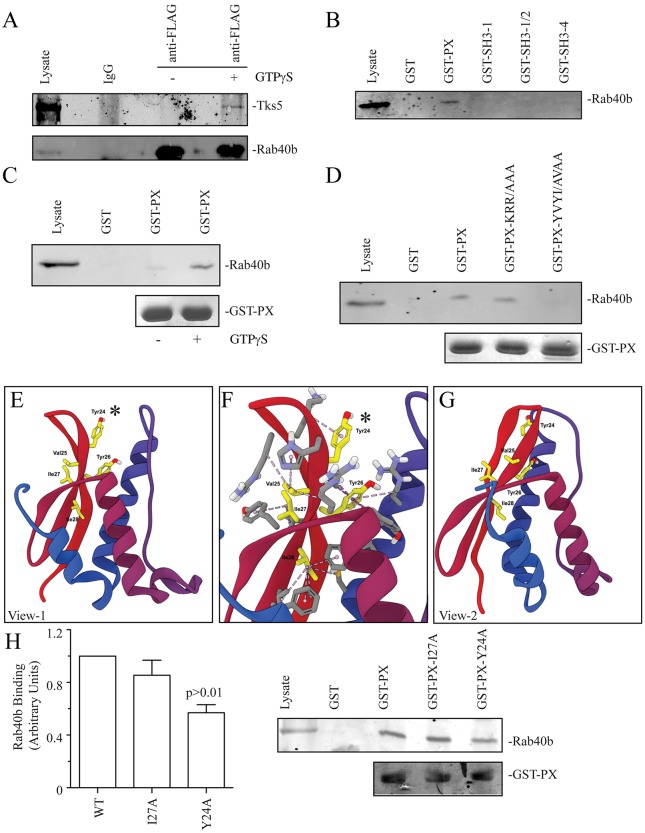
**Rab40b binds Tks5-PX domain.** (A) MDA-MB-231 cells stably expressing FLAG–Rab40b were lysed and lysates incubated in the absence or presence of GTPγS. FLAG–Rab40b was then immunoprecipitated using anti-FLAG antibody and immunoblotted with an anti-Tks5 antibody. Purified non-specific mice IgG was used as a control in immunoprecipitation experiments. (B) Glutathione bead pulldown assays with various GST-tagged Tks5 fragments. (C) GST–Tks5-PX (GST-PX)-coated glutathione beads were incubated with MDA-MB-231 lysates in the presence or absence of GTPγS. The amount of bound FLAG–Rab40b was then analyzed by immunoblotting with anti-FLAG antibodies. (D) Glutathione bead pulldown assays with various Tks5-PX mutants. (E–G) Models of PX domain structure depicting the position of residues and hydrophobic interactions within the PX domain. E and G show PX domains from different sides; F shows a magnified view of E that includes the 23-YVUI-28 motif. The asterisk highlights Tyr24, which is required for Rab40b binding. (H) The effect of Y27A and Y24A mutations on the ability of GST–PX to bind FLAG–Rab40b. Data shown are the mean±s.d. derived from three independent experiments.

To confirm that Tks5 interacts with Rab40b, we immunoprecipitated FLAG–Rab40b from MDA-MB-231 cells expressing FLAG–Rab40b (MDA-MB-231-FLAG-Rab40b) (Fig. 4A). Given that Rab GTPases cycle between GDP-bound inactive and GTP-bound active forms, the immunoprecipitation was done in the presence or absence of GTPγS. Consistent with Tks5 functioning as a Rab40b effector protein, Tks5 immunoprecipitated with FLAG–Rab40b in the presence of GTPγS (Fig. 4A).

Tks5 is a scaffolding factor that is known to bind to membranes through its PX domain, while interacting with various actin cytoskeleton regulators through its tandem SH3 domains (Fig. S3B). To identify the Tks5 domain that binds to Rab40b, we generated several GST-tagged Tks5 fragments, which included the PX and several different SH3 domains (Fig. S3B). To test the interaction of these fragments with Rab40b, we then performed glutathione bead pulldown assays using MDA-MB-231-FLAG-Rab40b cell lysates. Surprisingly, Rab40b did not bind to any of the SH3 domains tested, but rather interacted with the Tks5-PX domain (Fig. 4B). Furthermore, Tks5-PX and FLAG-Rab40b binding was GTP dependent (Fig. 4C), confirming that Tks5 is a canonical Rab40b effector protein.

The PX domain is a lipid-binding domain that is present in many proteins (Ellson et al., 2002). PX domains bind to phosphatidylinositols, thus mediating site-specific binding to lipids. Our data suggests that in addition to recruiting Tks5 to the invadopodia precursor at the plasma membrane, the Tks5-PX domain has a second function of binding Rab40b. Structural studies have shown that the PX domains consist of three β-sheets and three α-helices (Fig. S3C) (Psachoulia and Sansom, 2009). It was shown that α-helices form the phosphatidylinositol-binding pocket and are buried within the membrane bilayer (Psachoulia and Sansom, 2009). The parts of the PX that are exposed to the cytosol are the β-sheets, suggesting that these regions contain a Rab40b-binding motif. Thus, we next analyzed the amino acid sequence of Tks5-PX β-sheets looking for a potential Rab40b-binding site using two main criteria to identify possible Rab40b-binding sites. First, these sites had to be conserved among vertebrates. Second, these sites had to have a cluster of charged or hydrophobic amino acids. This *in silico* analysis led to the identification of two putative Rab40b-binding sites: 14-KRR-19 and 23-YVYI-28 (Fig. S3C). Next, we mutated these sites and tested their binding to FLAG–Rab40b. As shown in Fig. 4D, mutation of 23-YVYI-28 to 23-AVAA-28 disrupted the interaction of FLAG–Rab40b with Tks5-PX. We next generated a Tks5-PX structure model based on known PX domain structures (Fig. 4E–G). Importantly, with the exception of Y24 and I27, the other residues in the YVYI motif are buried within the PX domain core and participate in extensive hydrophobic interactions with other PX domain amino acid residues (Fig. 4E–G; Fig. S3D). Thus, it is unlikely these residues can mediate Tks5-PX binding to Rab40b. In contrast, Y24 and I27 face the cytosolic surface of PX domain. Therefore, we next mutated Y24 and I27 individually and tested the ability of the mutant to bind Rab40b. As shown in Fig. 4H, the Y24A mutation, but not I27A, is sufficient to disrupt the interaction between the PX domain of Tks5 and Rab40b.

### miR-204 regulates Rab40b and Tks5 expression in breast cancer cells

Thus far, our data have established that Rab40b is required for MMP2 and MMP9 targeting to the invadopodia and for breast cancer metastasis *in vitro* and *in vivo*. Thus, if Rab40b mediates breast cancer metastasis, Rab40b would be expected to be upregulated in breast tumors. To test this hypothesis, we analyzed Rab40b expression using publicly available patient breast tumor expression data (NCBI GEO dataset GSE58212). As shown in Fig. S4A, Rab40b is upregulated in grade 3 breast cancers, but its close homologues Rab40a and Rab40c are not. Additionally, Rab40b is also highly expressed in basal subtype (Fig. S4B). These data are consistent with our hypothesis that Rab40b is required for breast cancer cell invasion.

**Fig. 5.**
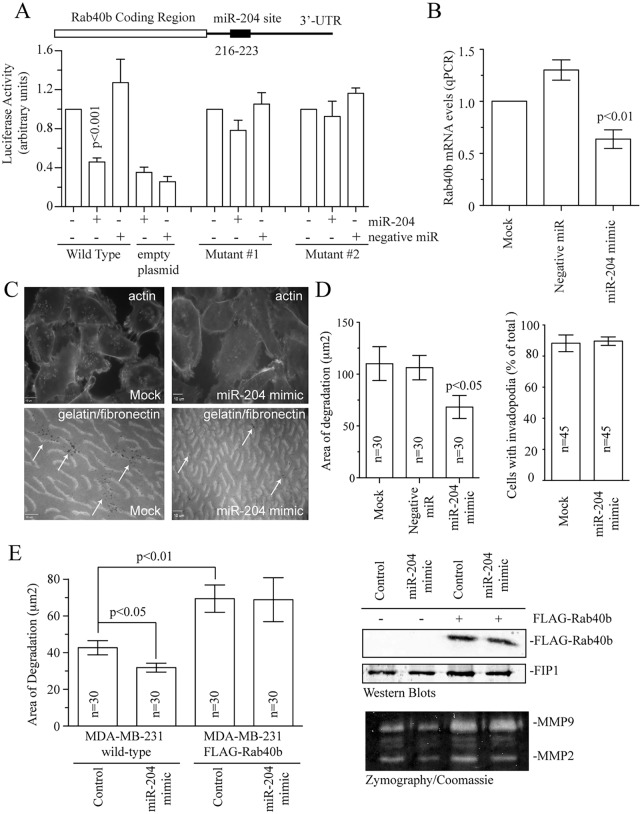
**miR-204 regulates Rab40b levels in breast cancer cells.** (A) The Rab40b-3′UTR contains a single putative miR-204 seed region. Wild-type and mutated Rab40b-3′UTRs were cloned into psiCheck2 dual luciferase reporter vector and cells were co-transfected with miR-204 mimic or negative control. Empty psiCheck2 plasmid was also used as a negative control. The activities of *Renilla* and firefly luciferase were analyzed 24 h after transfection. The data represent the percentage change in the ratio of *Renilla*:firefly activities compared with the controls. Data shown are the mean±s.d. from three independent experiments. Details of the two mutants is given in Fig. S4D. (B) Cells were mock-treated (without mimic) or treated with negative control or 50 nM of miR-204 mimic for 72 h. Cells were then harvested and levels of Rab40b mRNA analyzed by qPCR. Data shown are the mean±s.d. from 3 independent experiments. (C,D) Untreated MDA-MB-231 cells (control or mock) MDA-MB-231 cells treated with negative control or miR-204 mimic were plated on coverslips coated with gelatin supplemented with HiLyte-Fluor-488-labeled fibronectin. After 72 h of incubation, cells were fixed and stained with Rhodamine–phalloidin. Dark spots mark the sites of invadopodia-dependent ECM degradation (arrows). Top (actin) and bottom (gelatin with fibronectin) images for control and miR-204 mimic are of the same optical field. Data shown on bar graph (D) are the mean±s.d. from three independent experiments. *n* is the total number of individual cells analyzed. (E) Quantification of ECM degradation and MMP2 and MMP9 secretion in wild-type or FLAG–Rab40b-expressing MDA-MB-231 cells treated with miR-204 mimic and negative control. Data shown are the mean±s.e.m. of three independent experiments. *n* is the total number of cells analyzed for every treatment. The western blot shows the FLAG–Rab40b expression levels. FIP1 is used as loading control. The bottom gel shows the effects of miR-204 treatment and FLAG–Rab40b overexpression on secretion of MMP2 and MMP9 as measured by zymography assays. *P*-values given were compared to control and calculated with a two-tailed Student's *t*-test.

Although our findings demonstrate that Rab40b mediates breast cancer invasion it remains unclear how the expression of Rab40b is controlled. Recently, it was suggested that Rab40b is regulated by microRNA miR-204 (Li et al., 2011). Importantly, miR-204 is a well-established tumor-suppressor microRNA that is deleted in many cancers and is known to block metastasis (Imam et al., 2012). miR-204 levels are also decreased in grade 3 metastatic tumors (Fig. S4A,B). Thus, all these data demonstrate that miR-204 might be an important regulator of cancer cell metastasis. Given that it remains unclear how miR-204 affects the cancer invasion machinery, we decided to investigate whether miR-204 inhibits MMP2 and MMP9 targeting to invadopodia by decreasing cellular levels of Rab40b. *In silico* analysis identified a single miR-204 site within the 3′UTR of Rab40b (Fig. 5A; Fig. S4D). To test whether this site is necessary for miR-204 effect on Rab40b expression, we performed luciferase assays using full-length Rab40b 3′UTR fused to a luciferase reporter. Treatment of cells with a miR-204 mimic resulted in decreased luciferase activity (Fig. 5A). In contrast, mutation of the miR-204 seed region blocked the effect of miR-204 mimic on luciferase activity confirming that miR-204 acts by binding to a miR-204 site (216–223) within the Rab40b 3′UTR (Fig. 5A; Fig. S4D). To further confirm that Rab40b is a miR-204 target, we treated MDA-MB-231 cells with miR-204 mimic and analyzed the levels of endogenous Rab40b mRNA by quantitative real-time PCR (qPCR). As shown in Fig. 5B, miR-204 mimic significantly decreased endogenous Rab40b expression. Similar results were observed in other breast cancer cell lines tested, such as BT549 and SkBr3 (Fig. S4E).

Given that miR-204 decreases cellular levels of Rab40b in breast cancer cells, miR-204 would also be expected to inhibit MMP2 and MMP9 targeting to the forming invadopodia. To determine the role of miR-204 regulation of Rab40b levels in invadopodia formation and function, we treated cells with miR-204 mimic and assayed for matrix degradation activity using *in situ* zymography assays. Reduction of Rab40b levels by miR-204 led to decreased degradation of ECM associated with invadopodia, while having no effect on the cells ability to form invadopodia (Fig. 5C,D). Note, that the effect of miR-204 treatment on invadopodia formation and function was not as strong as Rab40b knockdown. This is likely due to the fact that miR-204 only led to an ∼30% decrease in Rab40b levels, whereas Ran40b KD1 lines had over 80% depletion of Rab40b. Overexpression of FLAG–Rab40b in MDA-MB-231 cells increased ECM degradation (Fig. 5E). Importantly, FLAG–Rab40b-induced matrix degradation was not affected by miR-204 treatment because the FLAG–Rab40b construct lacks the 3′UTR (Fig. 5E).

To confirm that miR-204 directly affects MMP2 and MMP9 secretion, we next analyzed the amount of MMP2 and MMP9 secreted into medium by control or FLAG–Rab40b-expressing MDA-MB-231 cells that were treated with miR-204 mimic. As shown in Fig. 5E (see bottom zymography blot), miR-204 treatment decreased secretion of MMP2 (miR-204 mimic was 46.3±4.5% of control set at 100%) as well as MMP9 (miR-204 mimic was 52.3±5.8% of control set at 100%; data are mean±s.d. calculated from three independent experiments) in the medium. In contrast, treatment of FLAG–Rab40b-expressing cells with miR-204 did not have any effect on MMP2 and MMP9 secretion (Fig. 5E). Importantly, overexpression of FLAG–Rab40b by itself increased secretion of MMP2 and MMP9 (Fig. 5E). Taken together, all these data demonstrate that miR-204 binds specifically to the Rab40b 3′UTR, thus decreasing cellular levels of Rab40b and thereby regulating MMP2 and MMP9 secretion as well as invadopodia-associated ECM remodeling.

**Fig. 6.**
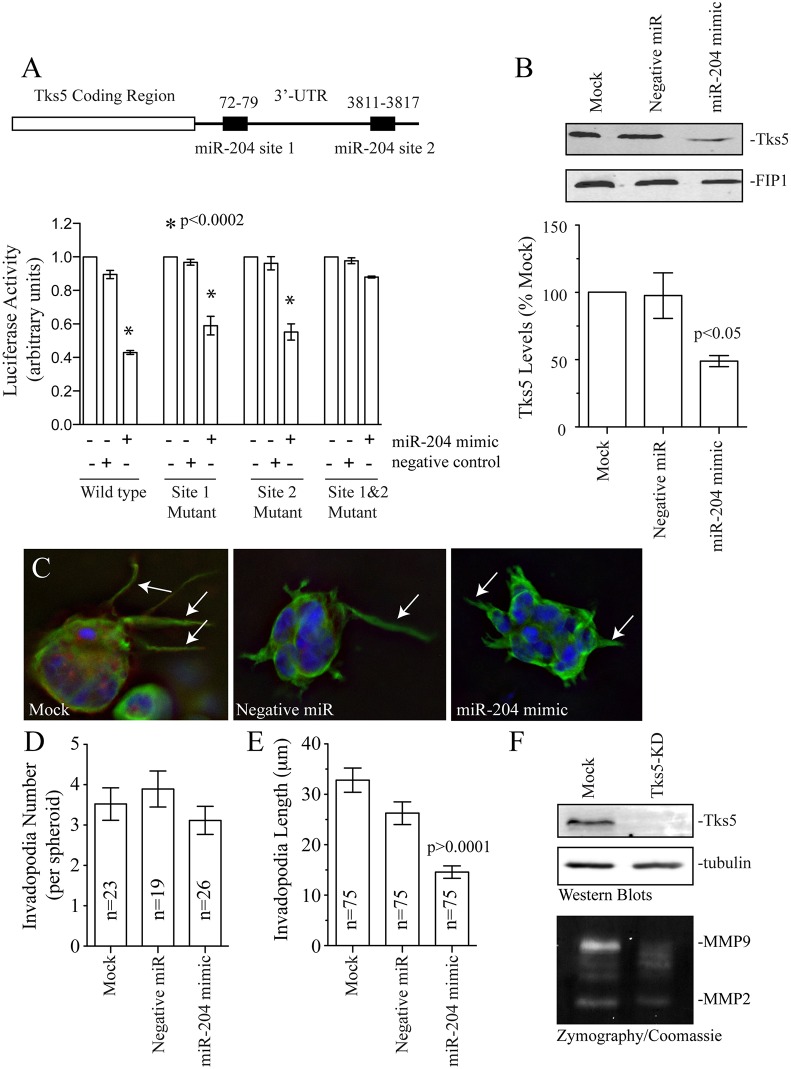
**miR-204 regulates Tks5 levels in breast cancer cells.** (A) The Tks5-3′UTR contains two putative miR-204 seed regions. 3′UTR fragments containing both the miR-204 seed regions were cloned adjacent to each other into psiCheck2 dual luciferase reporter vector and co-transfected with miR-204 mimic or negative control into cells. Data shown are the mean±s.d. from three independent experiments. (B) Western blot of MDA-MB-231 cells treated with miR-204 mimic or negative control, were probed with anti-Tks5 antibody. FIP1 is used as a loading control. The bar graph shows levels of Tks5 as a percentage of mock (untreated). Data shown are the mean±s.d. of three independent experiments. (C–E) MDA-MB-231 cells treated with miR-204 mimic or negative control miR, were embedded in a Matrigel+collagen I mixture and incubated for 4 days. The spheroids were then fixed and stained with anti-tubulin antibody to quantify the number and length of invadopodia (arrows). Data shown are the mean±s.d. of three independent experiments. *n* is the total number of spheroids analyzed. (F) The effect of Tks5 knockdown (Tks5-KD) on MMP2 and MMP9 secretion. The western blots show the extent of Tks5 knockdown. Tubulin is used as loading control. The bottom gel shows the effect of Tks5 depletion on secretion of MMP2 and MMP9 as measured by zymography assays.

One of the key properties of microRNAs is the ability to regulate multiple target proteins. To test whether miR-204 might affect other invadopodia and proteins regulating MMP2 and MMP9 transport, we used *in silico* analysis (using TargetScan analysis) to search for other putative miR-204 targets. Importantly, we identified two putative miR-204 seed regions within Tks5 3′UTR (Fig. 6A; Fig. S4D). To test whether any of these miR-204 sites regulate Tks5 levels, we again used luciferase assays. Treatment of cells containing the luciferase construct with both miR-204 sites from Tks5 3′UTR led to a decrease in luciferase activity (Fig. 6A). Mutating each seed region separately did not abolish the effect of miR-204 mimic on luciferase activity (Fig. 6A; Fig. S4E). However, mutating both the first and second seed regions blocked the effect of miR-204 mimic, thus indicating that miR-204 regulates Tk5 by binding to both microRNA sites (Fig. 6A). Furthermore, treatment of MDA-MB-231 or BT549 cell lines with miR-204 mimic resulted in a decrease of endogenous Tks5 by ∼50%, the extent of the depletion that is typically observed as the result of microRNA treatment (Fig. 6B; Fig. S4F).

Our aforementioned data shows that Rab40b and Tks5 are both miR-204 targets. Thus, it is likely that miR-204 inhibits cancer metastasis, at least in part, by blocking invadopodia-dependent ECM degradation and invadopodia extension. To test this hypothesis, we analyzed the effect of miR-204 mimic on invadopodia formation using MDA-MB-231 cells embedded in a Matrigel–collagen-I matrix (Fig. 6C,D; also see Figs 1, 2). Whereas treatment with miR-204 mimic did not affect invadopodia number, it resulted in a significant decrease in invadopodia length (Fig. 6C,D). Although Tks5 has been shown to be required for invadopodia maturation, it remains to be shown whether Tks5 directly mediates MMP2 and MMP9 secretion. To test that, we generated a MDA-MB-231 cell line stably expressing Tks5 shRNA (Fig. 6F). As shown in the Fig. 6F, Tks5 depletion inhibited secretion of MMP2 (Tks5-KD=21.3±3.5% of control set at 100%) and MMP9 (Tks5-KD=19.3±2.2% of control set at 100%; data are mean±s.d. calculated from three independent experiments). Taken together, all these data suggest that miR-204 inhibits cell invasion and migration by decreasing the levels of Rab40b and Tks5 and thus affecting invadopodia-associated ECM degradation (Courtneidge et al., 2005; Jacob et al., 2013; Sharma et al., 2013).

## DISCUSSION

Even though metastasis is the leading cause of mortality in cancer patients, the cellular and molecular events that occur during this process are not completely understood. MMPs have been shown to be important for tumor progression and metastasis and, therefore, have been considered for cancer therapy for many years. Therefore, it has been necessary to identify molecules involved in specific transport pathways that allow targeted secretion of MMPs. Although MMP2 and MMP9 have been shown to be upregulated in many cancers and enriched at the invadopodia, there are no defined pathways that describe how these enzymes are transported to specific ECM degradation sites that facilitate metastasis.

We had previously identified Rab40b as a protein required for targeted secretion of MMP2 and MMP9, and ECM degradation *in vitro* (Jacob et al., 2013). Even though traditional invasion assays allowed us to establish that Rab40b is involved in MMP2 and MMP9 targeting *in vitro*, the role of Rab40b in relation to invadopodia maturation and ECM degradation in a complex 3D system remained unclear. A 3D matrix provides the physical space for invadopodia maturation and allows cells to invade through a environment that is more like that *in vivo*. Indeed, several recent studies have shown that invasive protrusion formation in a 3D environment can be different from the ones observed in cells plated on 2D flat surfaces. Therefore, in this study, we focused on understanding the function of Rab40b in 3D experimental systems and mouse xenograft models. We have shown that Rab40b is present in invadopodia precursors suggesting an involvement of Rab40b in invadopodia initiation. Rab40b is mostly enriched at the tips of mature invadopodia, indicating that Rab40b is important for invadopodia maturation through active targeting of vesicles containing cargo like MMP2 and MMP9 to the invadopodial tips, from where MMP2 and MMP9 are released for focal ECM degradation. Degradation of the ECM not only provides space for invadopodia growth, but also results in release and/or activation of various growth factors required for angiogenesis, tumor growth and metastasis (Kalluri, 2003; Yurchenco, 2011). Thus, Rab40b-dependent MMP2 and MMP9 targeting to invadopodia is important in several steps during invadopodia formation and maturation *in vitro*.

In this study, we also analyzed the role of Rab40b in primary tumor metastasis *in vivo* using a mouse mammary fat pad xenograft model. Consistent with the involvement of Rab40b in cancer cell metastasis, our results show a significantly smaller number of micro-metastases in Rab40b KD lungs compared to control. Surprisingly, we also found that Rab40b knockdown resulted in a marked inhibition of primary tumor growth. At least to some extent, the smaller primary tumors after Rab40b knockdown could be caused by aberrant vasculogenesis, as characterized by smaller blood vessels. Tumors rely heavily on angiogenesis to expand their growth and metastasis potential. Interestingly, both MMP2 and MMP9 have been implicated in angiogenesis *in vitro* (Schnaper et al., 1993; Seftor et al., 2001) and *in vivo* (Itoh et al., 1998; Vu et al., 1998). Although the mechanism of how MMP2 and MMP9 contributes to angiogenesis remains obscure, the release of VEGF from the ECM due to the degradation activity of these gelatinases is one possible pathway. Upon depletion of Rab40b, the lack of MMP2 and MMP9 targeting could lead to reduced angiogenesis, thus inhibiting tumor growth. Consistent with that, in large tumors we could see increased apoptosis, which could be attributed to reduced angiogenesis, which deprives the tumors of nutrients and oxygen leading to cell death. Another possible explanation for the reduced tumor size in the Rab40b KD mice could be the lack of invasion and dispersal of cells within the tumor. Recently, it has been shown that the short-range dispersal or invasion ability of tumor cells within the tumor affects size, shape and growth rate of primary tumors (Waclaw et al., 2015). In our study, we have established that Rab40b is required for invadopodia formation and function. Therefore, in Rab40b KD mice, the inability of the cell to make invadopodia and degrade its surrounding environment could be affecting the movement of cancer cells within the tumor, resulting in smaller tumor size.

The dissemination of cancer cells from the primary tumor is associated with invadopodia formation and extracellular matrix degradation. Our results from mice killed at 8 weeks post injection show significantly smaller number of disseminated human cells in Rab40b KD lungs compared to control. However, in this time-matched study, we were unable to tease apart the direct effect of Rab40b on metastasis given that it also affects primary tumor growth. In order to circumvent the effect of Rab40b on tumor size, we analyzed metastasis in lungs from mice with size-matched large tumors over an extended time and found that there was no noted difference in number of disseminated human cells (Rab40b KD, 31±3.3 cells; control, 20±6.6 cells; mean±s.e.m.). However, whereas control mice were killed at about 8 weeks post-injection (due to maximum allowed tumor burden), most Rab40b-KD1 tumors were allowed to grow for 12 weeks to ensure that they reached a similar size to controls. Given that metastasis depends on tumor size as well as time, it is likely that the additional 4 weeks allowed Rab40b-KD tumors to catch up with their control counterparts with respect to metastasis. Taken together, our data suggest that Rab40b decreases tumor growth and metastasis, potentially as a direct result of cancer cell invasion as well as effects on primary tumor angiogenesis.

**Fig. 7.**
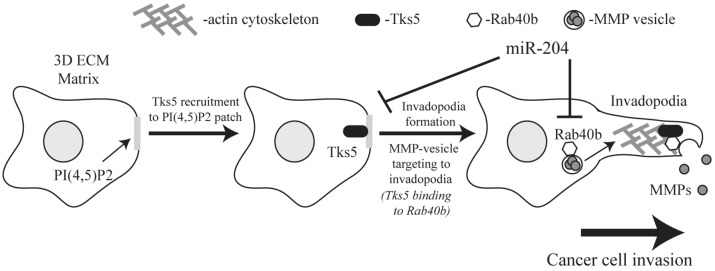
**Model for Rab40b mediated MMP2 and MMP9 targeting to the invadopodia.** Tks5 initiates invadopodia formation at PI(3,4)P_2_-enriched regions of the plasma membrane and acts as a tethering factor to recruit Rab40b vesicles containing MMP2 and MMP9. The interaction between Rab40b and Tks5 allows for the release of MMP2 and MMP9 in the ECM at invadopodia. miR-204 acts as a tumor suppressor by regulating both Rab40b- and Tks5-mediated invadopodia formation and function.

Another main objective of this study was to identify the mechanisms governing Rab40b function. Whereas the involvement of Rab40b in MMP targeting is becoming clearly established, how Rab40b ensures targeting of MMP2 and MMP9 transport vesicles to the tip of extending invadopodia remains completely unclear. All Rab GTPases function mainly by binding to various effector proteins. Consequently, identification of Rab effector proteins is an important step in understanding Rab function. Thus, we used proteomic analysis to identify Tks5 as a Rab40b effector protein (Fig. 7). Importantly, previous reports have established that Tks5 is a scaffold protein that is required for invadopodia formation and maturation (Blouw et al., 2008; Courtneidge et al., 2005). Thus, Tks5 makes an intriguing Rab40b-binding partner by mediating Rab40b targeting to the invadopodia plasma membrane to release transport vesicles containing cargo like MMP2 and MMP9.

Tks5 is a large scaffolding protein with a PX domain and five SH3 domains. The PX domain is generally known for its ability to bind to phosphatidylinositol lipids (Oikawa and Takenawa, 2009; Seals et al., 2005). Additionally, it has been suggested that phosphatidylinositol 3,4-bisphosphate [PI(3,4)P_2_]-enriched regions of the plasma membrane recruit Tks5 through its PX domain, thus initiating invadopodia formation (Sharma et al., 2013). Surprisingly, our data showed that Rab40b also binds to the PX domain of Tks5, suggesting that the Tks5-PX domain plays a dual role of targeting Tks5 to the site of invadopodia formation as well tethering Rab40b transport vesicles (Fig. 7). Structural studies of PX domains have shown that the α-helices that form the phosphatidylinositol-binding pocket are usually buried within the membrane bilayer, whereas the β-sheets are exposed to the cytosol (Psachoulia and Sansom, 2009). Based on these data, we hypothesized that these β-sheets might contain Rab40b-binding motifs. Consistent with this hypothesis, we show that Y24 is located within the second Tks5-PX β-sheet and is required for Rab40b binding.

Using both *in vitro* and *in vivo* models, we demonstrate that Rab40b and its effector protein Tks5, regulates MMP2 and MMP9 targeting and secretion during cell invasion and tumor growth (Fig. 7). Next, we used publicly available microarray data to analyze Rab40b expression in various breast cancers. Importantly, we discovered that Rab40b mRNA expression was increased in basal breast cancer as well as advanced stages of all breast cancer subtypes. Taken together, these data are consistent with the involvement of Rab40b during breast cancer cell invasion. However, it is unclear how cellular levels of Rab40b are regulated and why Rab40b expression is increased in metastatic cancers. Recently, it has been suggested that Rab40b might be a miR-204 target (Li et al., 2011). miR-204 is frequently lost in multiple cancers (Imam et al., 2012) and has been shown to act as a tumor suppressor in gliomas, colorectal and gastric cancers (Liu et al., 2015; Xia et al., 2015; Yin et al., 2014). Furthermore, decreased expression of miR-204 has been reported to correlate with a poor prognosis in breast cancer patients (Li et al., 2014), although how miR-204 suppresses tumor metastasis remains to be understood. Here, we have described a new mechanism by which miR-204 acts on breast tumorigenesis and metastasis by demonstrating that Rab40b and its binding proteinTks5 are both direct targets of miR-204 (Fig. 7). Consistent with this, we also show that miR-204 suppresses MMP2 and MMP9 secretion and invadopodia-associated ECM degradation.

As a whole, our data firmly establish Rab40b as a major regulator of targeted MMP2 and MMP9 secretion and breast tumor growth and metastasis *in vivo* and *in vitro*. Furthermore, this study also defines a new metastasis-regulating pathway that involves Rab40b and Tks5. Finally, we have shown that cellular levels of both Rab40b and Tks5 are regulated by the known tumor-suppressor microRNA miR-204. However, many questions remain. It is still unclear whether Rab40b also regulates MMP2 and MMP9 targeting in other cancers or whether it is limited to breast cancer. Additionally, whereas Rab40b regulates targeting of MMP2 and MMP9, it is unknown whether Rab40b regulates transport of other MMPs and proteases. At least transport of one of these MMPs, MMP14, is not dependent on Rab40b vesicles (Jacob et al., 2013), although MMP2, MMP9 and MMP14 are all enriched at invadopodia (this work; and see Castro-Castro et al., 2016). Although it is clear that MMP2 and MMP9, and MMP14 are transported by two different pathways, it is unclear whether these pathways converge at a later stage for efficient delivery of all MMPs to the invadopodia. Interestingly, MMP2 has been shown to be activated by MMP14. Thus, it is possible that MMP2 and MMP9, and MMP14 targeting through different transport pathways might function as a ‘co-incidence detection’ system to ensure the fidelity of MMP-dependent ECM remodeling at the invadopodia. Alternatively, different cancers might preferentially use either the MMP2 and MMP9, or MMP14 targeting pathways.

Rab40b is a unique small monomeric Ras-like GTPase that contains a SOCS box domain, a unique feature attributed only to the Rab40 subfamily of proteins (Stein et al., 2012). Interestingly, the SOCS box motif in other proteins has been implicated in regulation of cytokine secretion (Piessevaux et al., 2008) and have also been shown to bind cullin–elongin complex, thus mediating degradation of specific target proteins. Given that ubiquitin-dependent protein degradation has emerged as an important modulator of invadopodia and cancer metastasis, the identification of Rab40b-SOCS-interacting proteins might shed more light on the mechanisms that regulate MMP2 and MMP9 targeting during cancer cell invasion.

## MATERIALS AND METHODS

### Antibodies and constructs

Anti-FLAG (cat. no H-76, 1:100) and anti-tubulin (cat. no T5168, 1:100) antibodies were purchased from Sigma (St Louis, MO). Anti-CD31 antibody was from Abcam (cat. no ab28364, 1:100). Rabbit anti-Tks5 was made in-house using purified recombinant GST–Tks5 protein. It was affinity purified and used for western blotting at 1:250. Rabbit anti-FIP1 antibody was made by using purified recombinant human GST–FIP1. Antibody was used for western blotting at 1:1000. Alexa Fluor 594 and Alexa Fluor 488 conjugated to anti-rabbit-IgG and anti-mouse-IgG secondary antibodies were purchased from Jackson ImmunoResearch (West Grove, PA). Alexa-Fluor-568–phalloidin was purchased from Life Technologies (Grand Island, NY). Collagen I solution was purchased from Corning (Corning, NY). SB3CT (an MMP2 and MMP9 inhibitor) was purchased from Calbiochem (Billerica, MA). Matrigel was purchased from BD Biosciences (San Jose, CA). MiR-204 mimic was purchased from Ambion Life Technologies (Grand Island, NY).

### Cell lines

All cell lines were cultured as described previously (Jacob et al., 2013). MDA-MB-231 cell line stably expressing FLAG–Rab40b was created by cloning Rab40b into lentiviral pCS2-FLAG vector obtained from Addgene (Cambridge, MA). Stable knockdown and control MDA-MB-231 lines were generated using two Sigma lentiviral Rab40b shRNAs [TRCN0000047529 (KD1) and TRCN0000047530 (KD2)] plasmids.

### GST–Rab40b affinity chromatography

Putative Rab40b-binding proteins were identified using a GST–Rab40b affinity column as described previously (Prekeris et al., 2000). Tandem mass spectra were analyzed via Sequest using a human, mouse and rat database concatenated to a randomized human, mouse and rat database. DTASelect was used to reassemble identified peptides into proteins. Identified proteins were filtered at a <5% false discovery rate. Proteins that were identified from GST–Rab40b eluate but were absent in GST-only eluate were considered as a candidate Rab40b-binding proteins and are listed in Fig. S3A.

### Inverse invasion assay

The inverse invasion assay with MDA-MB-231 cells was adapted from von Thun et al., 2012. In brief, gelatin and a 50 μg/ml fibronectin plug was made on the filter as described in the *in situ* zymography protocol below. The cells were allowed to invade towards a gradient of 20% fetal bovine serum (FBS) and 10% Nu serum for 5 days. The cells were stained with 4 µM Calcein for 60 min and imaged at 10-µm steps to a total distance of 180 µm. ImageJ software was used to quantify the number of cells in every 10-µm step image from 20 µm to 140 µm. For quantification, at least 40 cells from five different fields per treatment were counted. Data shown are mean±s.d. derived from at least three independent experiments.

### *In situ* zymography

The *in situ* zymography and matrix degradation assay was performed as described previously (Jacob et al., 2013). To quantify invadopodia formation and localized matrix degradation, ten randomly chosen fields were imaged for each experiment. A total of 260–330 cells were counted in at least three independent experiments. To measure the number of cells with invadopodia, cells were counted based on the presence of actin puncta and degradation spots seen underneath the cells within the cell boundaries. To measure the invadopodia-associated area of degradation, the areas lacking FITC-fibronectin fluorescence were measured using Intelligent Imaging Innovations (Denver, CO) three-dimensional rendering and exploration software. Only degradation areas associated within cell boundaries were analyzed.

### Luciferase assay

The Promega Dual Luciferase Reporter Assay was used. Data were expressed as a ratio of the *Renilla* luciferase activity to firefly luciferase activity. The mean±s.e.m. was calculated from three independent experiments. For Rab40b, the entire 3′UTR was cloned into the psiCheck-2 Luciferase vector. For Tks5, given that the 3′UTR is 8-kb long, two 1-kb pieces containing the miR-204 seed region were cloned adjacent to each other. Mutations in the predicted miR-204 seed regions of the 3′UTR constructs were generated according to the Stratagene QuikChange site-directed mutagenesis protocol.

### 3D spheroid assay

A 75:25 ratio of a Matrigel and collagenI mixture of 100 µl was made into which 12×10^3^ cells were added. The cell–Matrigel–collagen mixture was then spotted onto a 3D chamber slide. To each well, 500 μl of 1:1 regular medium and growth-factor-rich medium (DMEM+20% FBS +10% NuSerum) was added and incubated from 48 h to 6 days. The spheroids were fixed and stained with anti-tubulin and Alexa-Fluor-568–phalloidin antibodies. Cells were imaged and the number as well as length of invadopodia was quantified from three independent experiments.

### Zymography assays

Mock, Tks5 shRNA-expressing, FLAG-Rab40b-expressing or miR-204 mimic-treated MDA-MB-231 cells were incubated in the complete medium at 37°C. After 24 h of incubation, the medium was replaced with Opti-MEM (Invitrogen, Carlsbad, CA) and cells were incubated at 37°C for another 24 h. Opti-MEM was collected and cell lysates were harvested using PBS containing 1% Triton X-100. The levels of secreted MMP2 and MMP9 in Opti-MEM were then analyzed by zymography.

### Mouse mammary fat pad xenograft assays

At total of 30 8-week-old female hairless SCID mice were divided into three experimental groups with 10 mice per group. Two million log phase MDA-MB-231 cells (wild-type or stably expressing Rab40b shRNA #1 or Rab40b shRNA #2) were injected into the number four right and left intact mammary glands. Primary tumor growth was measured weekly using calipers. Once a total tumor burden of 2 cm^3^ was reached, the mice were killed. Some mice with ulcerated tumors had to be killed earlier than predefined set end point in compliance with the animal research regulations. Mammary tumors and lungs were harvested. One half of the tissues were flash frozen for qPCR analysis while the other half was fixed and paraffin embedded for histology and immunohistochemical (IHC) analysis.

For the time end-point study, 20 8-week-old mice were divided into four experimental groups with 5 mice per group (wild type, shRNA control, Rab40b shRNA#1 and Rab40b shRNA#2). The injections were done as described above and all mice were killed at week 8. Mammary tumors and lungs were harvested for qPCR, histology and IHC analysis. All animal experiments were performed according to approved guidelines.

### Breast cancer data mining

The data shown was obtained from the NCBI GEO dataset GSE58212. The dataset is an mRNA expression profiling of 283 breast cancer samples which was performed using the SurePrint G3 Human GE 8x60K one-color microarrays from Agilent (Agilent Technologies, Santa Clara, CA, USA). The data represented in the graph are shown as standard error of the mean.

### *In situ* analysis of lung metastasis

*In situ* FISH analysis was performed by the University of Colorado Denver Cytogenetics Core. Briefly, unstained slides with formalin-fixed, paraffin-embedded tissue sections were subjected to a dual-color FISH assay using the Human/Mouse probe set. This probe set was prepared by labeling 1 µg of Human Cot-1 DNA and Mouse Cot-1 DNA (Invitrogen) respectively with SpectrumRed and SpectrumGreen conjugated dUTPs (Abbott Molecular) using the Vysis Nick translation kit (Abbott Molecular), according to manufacturer’s instructions. The labeled DNAs were ethanol precipitated with herring sperm DNA as carrier (1:50) and each pellet was diluted to a final concentration of 50 ng/µl with tDenHybTM-2 hybridization buffer (Insitus Biotechnologies cat. no D102). The probe set was prepared such that it contained 100ng of each DNA per 4.5 µl and this volume was used on every 113mm2 hybridization area. The FFPE specimens were analyzed in an epifluorescence microscope using single interference filters sets for green (FITC), red (Texas red), blue (DAPI), and dual (red/green) and triple (blue, red, green) band pass. Each section was entirely examined under low magnification and selected areas were investigated under high magnification objectives.

### GST–Tks5 fragments and glutathione bead pulldown assay

All GST fusion proteins were generated as previously described (Willenborg et al., 2011). Three domains of Tks5 were cloned into pGEX-2T vector to make GST–Tks5 fragments; PX domain (amino acids 4–128), SH3-1 domain (amino acids 129–265), SH3-4 domain (amino acids 722–936). Glutathione bead pull downs were performed with 1% Triton X-100 lysates generated from MDA-MB-231 cells stably expressing FLAG–Rab40b. Lysates were incubated with 10 µg of various GST–Tks5 fragments. After incubation, FLAG–Rab40b bound to GST–Tks5 fragments was isolated using glutathione–Sepharose beads and washed in reaction buffer (20 mM Hepes pH 7.4, 150 mM NaCl, 1mM MgCl2 and 0.1% Triton X-100). Bound FLAG–Rab40b was eluted with 1× western running buffer containing 0.2% SDS. Samples were separated by SDS-PAGE and probed with anti-FLAG antibody.

### RT-PCR and quantitative PCR

Total RNA was extracted from 2×107 MDA-MB-231 cells using TRIzol (Invitrogen) according to the manufacturer's protocol. Reverse transcription to cDNA was performed with iScript Reverse transcription supermix for RT-qPCR. Quantitative PCR was performed using Taqman PCR master mix from Applied Biosystems (Grand Island, NY). To quantify the efficiency of knockdown, cDNA from mock- or siRNA-treated cells was analyzed in triplicate by qPCR amplification using Taqman Master Mix and an Applied Biosystems ViiA7 Real-Time PCR System. The qPCR amplification conditions were: 50°C (2 min), 95°C (10 min), 40 cycles at 95°C (15 s), 60°C (1 min). Taqman primers for Rab40b (Hs00895201_mH) and β-actin (Hs99999903_m1) control were purchased from Applied Biosystems (Grand Island, NY). Relative quantification was calculated by the ΔΔCT method. Data is shown as the fold change (averaged from three independent experiments) in Rab40b-knockdown cells compared with mock-treated cells.

### Statistical analysis

A two-tailed Student's *t*-test was used to determine statistical significance. In all cases data was collected from at least three independent experiments. In experiments where cells or spheroids were analyzed *n* represents total number of cells or spheroids analyzed for each condition. In all cases *P*≤0.05 was regarded as significant. The analysis was done using GraphPad Prism software.
